# Assessment of diagnostic utility of serum hemeoxygenase-1 measurement for acute exacerbation of interstitial pneumonias

**DOI:** 10.1038/s41598-022-17290-0

**Published:** 2022-07-28

**Authors:** Yuki Kata, Yu Hara, Yoichi Tagami, Aya Yabe, Kota Murohashi, Ryo Nagasawa, Kentaro Nakashima, Hiroaki Fujii, Yusuke Saigusa, Masafumi Shiida, Keisuke Watanabe, Nobuyuki Horita, Nobuaki Kobayashi, Takeshi Kaneko

**Affiliations:** 1 Department of Pulmonology, Yokohama City University Graduate School of Medicine, 3-9 Fukuura, Kanazawa-ku, Yokohama, 236-0004 Japan; 2 Department of Respiratory Medicine, Yamato Municipal Hospital, 8-3-6 Fukami-nishi, Yamato, 242-8602 Japan; 3 Department of Biostatistics, Yokohama City University School of Medicine, 3-9 Fukuura, Kanazawa-ku, Yokohama, 236-0004 Japan; 4 Research & Development Division, Minaris Medical Co., 600-1, Minami-ishiki, Nagaizumi-cho, Sunto-gun, Shizuoka, 411-0932 Japan

**Keywords:** Biomarkers, Medical research

## Abstract

The present study aimed to evaluate whether serum heme oxygenase (HO)-1 could be a reliable blood biomarker for diagnosing acute exacerbations (AEs) of both idiopathic interstitial pneumonia (IIP) and secondary interstitial pneumonia (SIP). Serum HO-1 levels of newly diagnosed patients with IP were measured, and the relationships between serum HO-1 and other serum biomarkers and high-resolution CT scores, were evaluated. Blood samples were collected from 90 patients with IIP, including 32 having an AE, and 32 with SIP, including 9 having an AE. The patients having an AE had significantly higher HO-1 levels than those not having an AE (35.2 ng/mL vs. 16.4 ng/mL; *p* < 0.001). On receiver operating characteristics (ROC) curve analysis for serum HO-1 ability to detect an AE, the area under the ROC curve (AUC) was 0.87 in patients with IIPs and 0.86 in those with SIPs. Also, in patients with both IIPs and SIPs, the combination of the serum HO-1 level and the GGO score showed favorable AUCs (IIPs: 0.92, SIPs: 0.83), though HO-1-not-including model (combination of LDH and GGO) also showed acceptable AUCs. Serum HO-1 could be a clinically useful biomarker for the accurate diagnosis of patients with AEs.

## Introduction

The prognosis of acute exacerbations (AEs) of interstitial pneumonia (IP) is poor, and the histological pattern typically involves diffuse alveolar damage (DAD) superimposed on lung fibrosis^1^. However, because the histological findings of AE of IP include not only DAD, but also other atypical subtypes including diffuse alveolar hemorrhage, organizing pneumonia, pulmonary thromboembolism, lung cancer, and bronchopneumonia, it can sometimes be difficult to distinguish between AE and non-AE IP in clinical practice^2,3^. In addition, there are some established biomarkers for diagnosing AEs.

Macrophage polarization plays key roles in all phases of wound healing, which are inflammation, proliferation, and remodeling (fibrosis), and the interaction between M1 and M2 macrophages derived from peripheral monocytes (uncommitted macrophages (M0)) is reported to be closely correlated with disease progression in patients with AEs of IP^4,5^. Heme oxygenase (HO)-1 is a 32-kDa heat shock protein that converts heme into carbon monoxide (CO), iron, and bilirubin, and is expressed exclusively on the anti-inflammatory M2 macrophage lineage, but not the pro-inflammatory M1 macrophage, by heat shock and oxidative stress conditions^6^. Furthermore, the anti-inflammatory and anti-oxidative actions of each product originating from HO-1, CO, and biliverdin sustain the properties of M2^7^. Prior research suggested that serum HO-1 measurement increased mainly in alveolar macrophages of patients with AEs of IP and contributed to detect DAD and predict disease prognosis in patients with IP^3,8–10^.

The purposes of the present research were to evaluate the utility of serum HO-1 for detection of AE in patients with each subtype, including secondary interstitial pneumonias (SIPs), as well as idiopathic interstitial pneumonias (IIPs), including the previously reported validation, and to compare detectability to other biomarkers commonly used in clinical practice.

## Materials and methods

The methods of this study including the patient’s recruitment, high-resolution CT (HRCT) scoring, the technique of serum HO-1 measurement followed those of our original research reported in the past^8–10^.

### Study patients and the diagnosis of IP

This study enrolled a total of 122 newly diagnosed and untreated IP patients who had been admitted to the hospital from 2011 to 2020. The extracted data included the patients’ medical histories, physical examination findings, results of blood biomarkers, and HRCT findings. The diagnosis of idiopathic pulmonary fibrosis (IPF) or idiopathic nonspecific interstitial pneumonia (iNSIP) was based on the established criteria^11–13^. Patients with non-IPF IIP who could not undergo surgical lung biopsy due to severe respiratory failure were diagnosed as unclassifiable IIP. The diagnosis of collagen vascular disease-related IP (CVD-IP) was confirmed by physical findings, serological testing, and HRCT findings that were consistent with IP. The diagnosis of drug-induced lung injury (DILI) was based on the previously reported criteria^14^. All of the enrolled patients were categorized into either of two groups: those with IP not having an AE, and those with IP having an AE. An AE was defined as significant respiratory deterioration including clinical worsening of dyspnea, hypoxemia, or the worsening or severe impairment of gas exchange characterized by new bilateral ground-glass opacification/consolidation superimposed on a background pattern consistent with IP pattern not fully explained by cardiac failure or fluid overload^1,15,16^. We also ruled out the infectious pneumonia based on the sputum and blood culture or clinical evidence that antimicrobials did not work. Finally, we categorized AE of IPF or iNSIP and unclassifiable IIP into AE of IIPs group and AE of CVD-IP into AE of SIPs group. The DILD patients were classified as a triggered AE of IIP. As the additional evaluation, we categorized IP not having AE into IP at the stable condition and acute respiratory worsening (ARW) having an alternative explanation such as infection, not requiring steroid pulse therapy. All methods were performed in accordance with the relevant guidelines and regulations.

### HRCT scoring

The HRCT findings were evaluated using the semiquantitative scoring method described by Ooi et al.^17^. The lungs were divided into six distinct zones, three on each side. Ground-glass opacity (GGO) and honeycombing on HRCT were then scored based on the percentage of disease extent in each of the 6 lung lobes. A global score was calculated by adding the scores for each abnormality in all lobes. HRCT was performed at hospitalization; each scan was independently assessed by three pulmonologists (HY, TY, and MK (experience-year: more than 10 years)).

### Serum HO-1 enzyme-linked immunosorbent assay (ELISA) measurement

Serum HO-1 levels were measured at the time of IP diagnosis for patients with IP without AE or at the time of AE diagnosis for patients with AEs of IP using the IMMUNOSET HO-1 (human) ELISA development set (Enzo, Farmingdale, NY, USA). The details of this ELISA method have been described previously^8^. Assay validation was performed based on the reproducibility of the ELISA standard curve for serum HO-1, the intra- and inter-assay tests, and the percentage recovery test. It was confirmed that all of these results were acceptable^8^.

### Other blood biomarker measurements

Blood samples were obtained at the same time as serum HO-1 measurement. Serum HO-1 was measured along with lactate dehydrogenase (LDH; normal < 225 U/L), surfactant protein (SP)-D (normal < 110 ng/mL), and KL-6 (normal < 500 U/mL).

### Statistical analysis

All data are expressed as medians with 25th to 75th percentiles unless otherwise noted. Statistical analysis was performed using JMP11 (SAS Institute, Inc., Cary, NC, USA) and R software, version 4.1.1 (R Foundation for Statistical Computing, Vienna, Austria). Group comparisons were performed using Wilcoxon’s rank-sum test or the chi-squared test. Non-parametric Spearman’s rank correlation coefficients were calculated to assess the correlations between the serum HO-1 levels and other clinical parameters. A receiver operating characteristic (ROC) curve analysis was performed to determine the most suitable cut-off levels of serum HO-1 and other blood biomarkers for detecting AEs. The predictive performance of the composite parameters including HO-1, LDH, and GGO score was investigated using ROC and the Delong method and the logistic regression model for AE was employed to assess the serum HO-1. *P* < 0.05 was considered significant.

### Study approval

All participants provided informed consent prior to participation in this research. All aspects of the study were approved by the Institutional Review Board of Yokohama City University Graduate School of Medicine (approval number B170900025). The authors conducted this research in full accordance with the Declaration of Helsinki.

## Results

### Patients’ characteristics

Clinical characteristics of patients with IP are summarized in Table 1. One hundred and twenty-two patients with IP, including IIPs (32 having an AE and 58 not having an AE) and SIPs (9 having an AE and 23 not having an AE), were evaluated. The IIP group included 49 IPF (41 diagnosed radiographically and 8 diagnosed with surgical lung biopsy (SLB) and 41 non-IPF IIPs patients including 8 iNSIP diagnosed with SLB. The remaining non-IPF IIPs were categorized as unclassifiable IIPs. The SIP group included 28 CVD-IP. In both IIP and SIP groups, serum HO-1 and GGO scores were significantly higher in patients having an AE than in those not having an AE. The serum HO-1 levels according to IP subtypes were shown in Supplement Table 1. Also, those not having an AE included 15 patients with an ARW due to 13 infection, 1 lung edema caused by renal failure, and 1 pneumothorax. Serum HO-1 was significantly higher than in patients having an AE than in those having an ARW or at the stable condition (Supplement Fig. 1).

**Table 1:** Patients’ characteristics.

Variable	IIPs		AE vs. no AE	SIPs		AE vs. no AE
	AE (*N* = 32)	No AE (*N* = 58)		AE (*N* = 9)	No AE (*N* = 23)	
Age (y)	77 (70.8–80.3)	76 (69.5–82)	0.671	72 (66–77)	69 (62–77)	0.442
Sex (Male)	26 (81)	48 (83)	0.760	3 (33)	7 (30)	0.712
CCIS	3 (2–5.5)	2 (1–3.5)	0.061	2 (1–3)	3 (1–5)	0.549
**IP subtype**			0.798			0.181
IPF	18 (56)	31 (53)				
Non-IPF IIPs	14 (44)	27 (47)				
CVD-IPs				9 (100)	19 (83)	
Others				0 (0)	4 (17)	
**Biomarkers**						
LDH, IU/L	276 (233–352)	202 (190–235)	< 0.001	265 (201–394)	246 (180–289)	0.367
SP-D, ng/mL	210 (90–325)	129 (88–263)	0.199	214 (153–291)	176 (149–212)	0.386
KL-6, U/mL	775 (341–1455)	617 (339–882)	0.281	1249 (657–2358)	905 (553–1956)	0.593
HO-1, ng/mL	34.3 (25.3–53.0)	15.5 (10.2–24.1)	< 0.001	41.8 (21.9–53.3)	17.1 (12.1–22.0)	0.002
HRCT scores						
Honeycomb	3 (0–8)	3 (0–6)	0.706	3 (0.5–7)	1 (0–4)	0.210
GGO	8 (7–11)	2 (2–4)	< 0.001	8 (4–17)	3 (2–5)	0.016

AE, acute exacerbation; CCIS, Charlson Comorbidity Index score; CVD, collagen vascular disease; GGO, ground-glass opacity; HO-1, heme oxygenase-1; HRCT, high-resolution computed tomography; IIPs, idiopathic interstitial pneumonias; IP, interstitial pneumonia; IPF, idiopathic pulmonary fibrosis; KL-6, Krebs von den Lungen-6; LDH, lactate dehydrogenase; SIPs, secondary interstitial pneumonias; SP-D, surfactant protein-D.

### Relationship between the serum HO-1 level and other parameters, diagnostic utility of biomarkers in AEs of IIPs and SIPs

Among patients with IIPs, there were significant correlations between serum HO-1 and serum LDH, SP-D, and KL-6 (*R* = 0.51, 0.55, and 0.30) levels, and between the serum HO-1 level and the GGO score (*R* = 0.46); however, there was no significant correlation of the HO-1 level with the honeycomb score. In addition, in patients with SIPs, there were significant correlations between the serum HO-1 and serum LDH levels (*R* = 0.57); however, there were no significant correlations of the HO-1 level with the other blood parameters and the HRCT score (Table 2).

**Table 2:** Relationships between the serum HO-1 level and other parameters.

	Variable	N	R	95%CI	*P* values
IIPs Serum HO-1	Serum LDH	90	0.51	0.34–0.65	< 0.001
	Serum SP-D	68	0.55	0.36–0.70	< 0.001
	Serum KL-6	84	0.30	0.09–0.48	0.006
	Honeycomb score	90	0.13	− 0.08–0.34	0.214
	GGO score	90	0.46	0.27–0.61	< 0.001
SIPs Serum HO-1	Serum LDH	32	0.57	0.28–0.77	< 0.001
	Serum SP-D	19	0.17	− 0.20–0.60	0.487
	Serum KL-6	28	− 0.17	− 0.48–0.19	0.374
	Honeycomb score	32	− 0.06	− 0.41–0.23	0.764
	GGO score	32	0.31	0.04–0.61	0.08

CI, confident intervals; GGO, ground-glass opacity; HO-1, heme oxygenase-1; IIPs, idiopathic interstitial pneumonias; KL-6, Krebs von den Lungen-6; LDH, lactate dehydrogenase; SIPs, secondary interstitial pneumonias; SP-D, surfactant protein-D.

ROC curve analyses for the serum HO-1 level and other parameters were performed to discriminate patients having an AE from patients not having an AE. Among patients with IIPs, the areas under the ROC curves (AUCs) of the serum HO-1 level, the LDH level, and the GGO score were high (0.87, 0.78, and 0.88, respectively). For serum HO-1, the best cut-off level was 22.8 ng/mL, and using this cut-off level, serum HO-1 had a sensitivity of 87% and a specificity of 74% for detecting an AE. In patients with SIPs, the AUCs of the serum HO-1 level and the GGO score were high (0.86 and 0.78, respectively). For serum HO-1, the best cut-off level was 20.5 ng/mL, and using this cut-off level, serum HO-1 had a sensitivity of 89% and a specificity of 74% for detecting an AE (Table 3).

**Table 3:** Diagnostic utility of biomarkers of AEs of IIPs and SIPs.

	Variable	N	AUC	Best cut-off values	Sensitivity, %	Specificity, %	*P* values
IIPs	Serum HO-1, ng/mL	90	0.87	22.8	87	74	< 0.001
	Serum LDH, U/L	90	0.78	233	78	76	< 0.001
	Serum SP-D, ng/mL	68	0.59	203	53	74	0.043
	Serum KL-6, U/mL	84	0.57	838	47	75	0.071
	Honeycomb score, points	90	0.52	8	28	82	0.773
	GGO score, points	90	0.88	6	84	85	< 0.001
SIPs	Serum HO-1, ng/mL	32	0.86	20.5	89	74	0.012
	Serum LDH, U/L	32	0.60	393	33	91	0.200
	Serum SP-D, ng/mL	19	0.63	207	63	73	0.440
	Serum KL-6, U/mL	28	0.43	2931	100	15	0.656
	Honeycomb score, points	32	0.64	6	33	91	0.129
	GGO score, points	32	0.78	5	78	83	0.008

AE, acute exacerbation; AUC, area under the receiver operating characteristic curve; GGO, ground-glass opacity; HO-1, heme oxygenase-1; IIPs, idiopathic interstitial pneumonias; KL-6, Krebs von den Lungen-6; LDH, lactate dehydrogenase; SIPs, secondary interstitial pneumonias; SP-D, surfactant protein-D.

### Diagnostic utility of composite parameters consisting of the serum HO-1 or LDH levels and the GGO score in AEs of IIPs and SIPs

**Figure 1:**
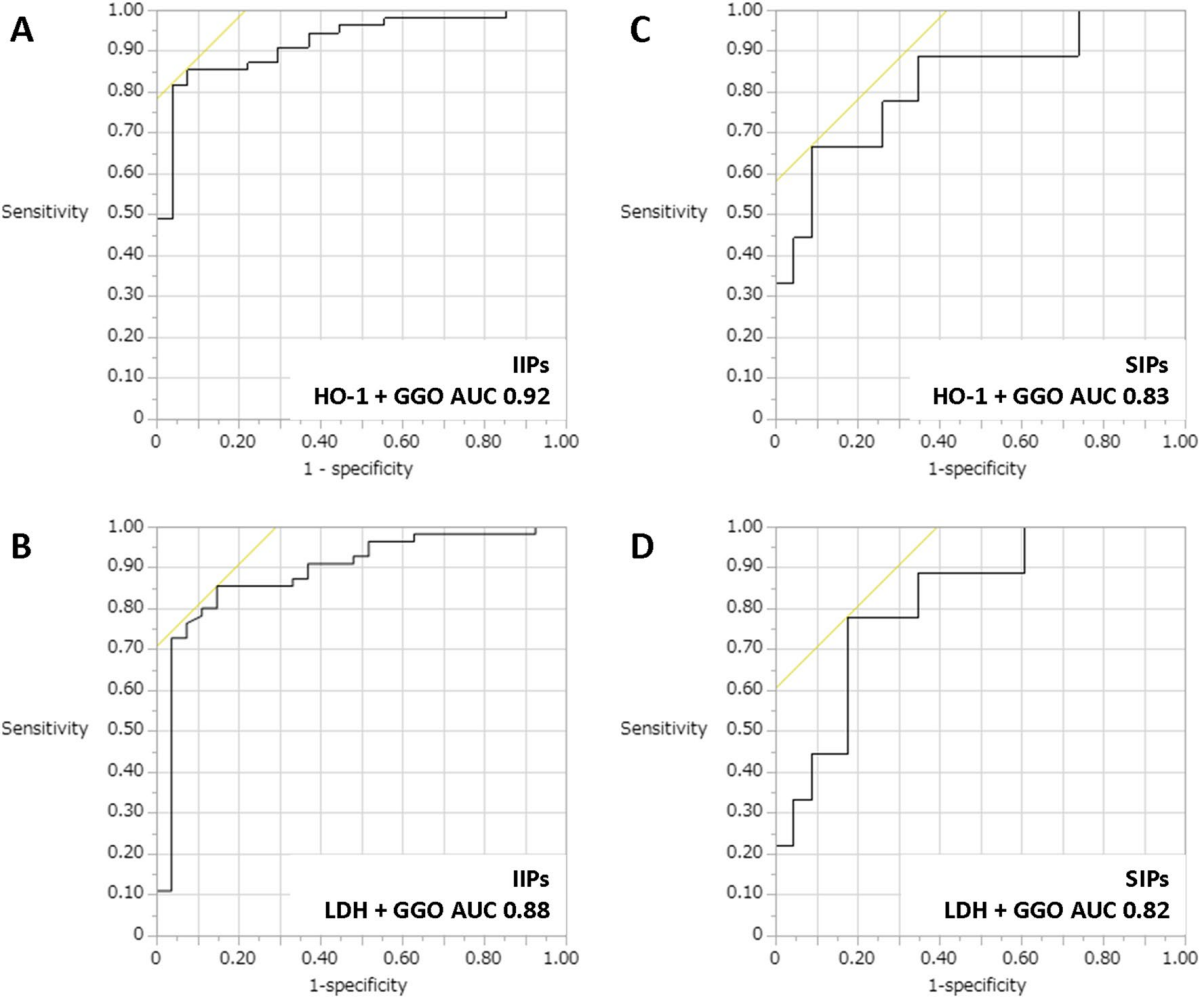
Diagnostic ulitity of the serum HO-1 or LDH levels and the GGO score for AEs. In patients with IIPs and SIPs, AE detectability was evaluated by a composite parameter combining serum HO-1 (IIPs: A SIPs: C), LDH level (IIPs: B SIPs: D), and GGO score. The combination of serum HO-1 values and GGO scores in both groups showed higher AUCs than the combined AUCs of serum LDH values and GGO scores.

From the results of Tables 2 and 3, we evaluated the predictive performance of serum HO-1, LDH, and GGO score. In both patients with IIPs and those with SIPs, the detectability of AE was evaluated using composite parameters, including the serum HO-1 (Fig. 1A for IIPs and (C) for SIPs) and LDH (Fig. 1B for IIPs and (D) for SIPs) levels and the GGO scores. In patients with both IIPs and SIPs, the combination of the serum HO-1 level and the GGO score showed favorable AUCs (IIPs: 0.92, SIPs: 0.83), though there were no significant differences between HO-1-including model and HO-1-not-including model using the Delong method. Also, from the logistic regression analysis including serum HO-1, LDH, and GGO score, serum HO-1 and GGO score proved to be significant in patients with IIPs, while only GGO score was significant in those of SIPs (Table 4).

**Table 4:** The multivariate logistic regression model for AE.

	Variable	Estimate (Odds ratio)	95%CI	*P* value
IIPs	Serum HO-1	1.06	1.01–1.11	0.006
	Serum LDH	1.00	0.99–1.01	0.768
	GGO score	1.42	1.16–1.75	< 0.001
SIPs	Serum HO-1	1.05	1.00–1.12	0.083
	Serum LDH	1.00	0.99–1.01	0.784
	GGO score	1.19	1.00–1.41	0.036

AE, acute exacerbation; CI, confident intervals; GGO, ground-glass opacity; HO-1, heme oxygenase-1; IIPs, idiopathic interstitial pneumonias; LDH, lactate dehydrogenase; SIPs, secondary interstitial pneumonias.

## Discussion

Oxidative/nitrosative stress caused by an imbalance between cellular production of reactive oxygen species/reactive nitrogen species and endogenous antioxidants such as stress response protein including HO-1 and classic antioxidant enzymes including superoxide dismutases, catalase, and glutathione peroxidase might play a major role in the progression of various lung diseases such as IPF, chronic obstructive pulmonary disease, and DAD^18–22^. On the other hand, macrophages play key roles in all phases of adult wound healing, which are inflammation, proliferation, and remodeling. Human peripheral monocytes are differentiated uncommitted macrophages (M0), and they are broadly polarized to pro-inflammatory M1 macrophages and anti-inflammatory M2 macrophages^23^. The interaction between M1 and M2 macrophages is reported to be closely correlated with disease progression in patients with IP, including AEs^24–26^. In patients with AEs of IP, HO-1 is strongly and exclusively induced on M2 macrophages, which differentiate in response to IL-4, IL-10, and IL-13 and produce large amounts of TGF-β1, resulting in extracellular matrix deposition, epithelial-mesenchymal transition, fibroblast activation, and cell death, depending on M1 macrophage activation^4,5,24,25^. Consistent with this previous research, we have demonstrated that serum HO-1 is useful for distinguishing between AE and stable IP, and serum HO-1 levels were positively correlated with serum levels of SP-D and the GGO score^9^. However, because this research included a very small number of cases, the clinical utility of serum HO-1 measurement has not been examined for each IP subtype (for each IIP and SIP); therefore, the ability of serum HO-1 to detect AEs in patients with each IP subtype was evaluated for the purpose of validation and to compare with the detectability to other biomarkers commonly used in clinical practice.

IP is characterized by alveolar inflammation leading to progressive fibrosis^27^. In the presence of alveolitis, surfactant apoproteins such as SP-A and SP-D are secreted by type II pneumocytes, and these apoproteins can be detected in the serum^27,28^. SP-A and SP-D concentrations are reported to correlate with the extent of alveolitis (denoted by HRCT findings of GGO), but not with the progression of fibrosis^29^. The serum LDH level is a non-specific biomarker that is elevated in various inflammatory diseases and reflects inflammation and cellular injury in the lungs of IP patients and has been mentioned as a prognostic factor in AE patients with IPF^30,31^. Consistent with this observation, it was found that the serum HO-1 level was positively correlated with the serum LDH and SP-D levels and the GGO score (especially in IIPs) in the present study. Therefore, we hypothesize that serum HO-1 as an M2 macrophage activation marker could provide a highly specific marker of alveolitis in patients having an AE. The points that serum HO-1 did not correlate with SP-D level or GGO score in SIPs were that the number of cases diagnosed with AE was much smaller than that of IIPs and that the included cases seemed to be more heterogeneous than those of IIPs. It was considered essential to verify only the SIPs, which increased the number of cases in the future.

AEs can occur in both groups of IIPs and SIPs, presenting with rapid respiratory failure, and the primary treatment is steroid pulse therapy^15,16,32^. AE diagnosis is often difficult, because the clinical manifestations of pulmonary infections, congestion, and thromboembolism are sometimes similar to those of AEs. In cases where a diagnosis is difficult, various drugs such as steroids, antibacterial drugs, and diuretics are administered in combination, resulting in unnecessary drug administration. Therefore, it is essential to improve the AE diagnosis rate. We have reported that, in 28 patients with IP, serum HO-1 levels helped predict the severity of the disease and hospital mortality and were higher in patients who developed AE than in those who did not^9^. In the present study involving a larger number of patients, the serum HO-1 level had a favorable AUC value similar with other biochemical biomarkers such as serum LDH in AE diagnosis, and a composite parameter consisting of serum HO-1 and the GGO score provided high AUC values in both IIP and SIP groups. Furthermore, although the number of cases was small, it was shown that serum HO-1 was significantly higher in AE than ARW due to infection, lung edema, and pneumothorax. Therefore, serum HO-1 measurement could contribute to providing an accurate diagnosis of patients with AEs, leading to rapid decision-making related to treatment, including steroid pulse therapy or other options such as antibiotics. Regarding the validation of the discrimination performance between AE and ARW, it was considered that a larger-scale prospective research was necessary.

Although the serum HO-1 level might have been shown to be a useful biomarker in patients with AE, there are several limitations in the present research. First, the number of patients was still small and from a single institution. The clinical diagnoses of the patients enrolled with SIPs were much smaller than those of IIPs and heterogeneous and no similar trends to IIPs could be found. The reproducibility of the findings of this study needs to be confirmed through validation cohorts which increased the number of cases in the future. Second, after the onset of AEs, a certain number of patients did not undergo histopathological evaluation because of severe respiratory failure. Therefore, the evaluation of serum HO-1 related to the degree of DAD and organizing pneumonia in affected lesions of the lung has not been investigated. Third, we have not evaluated the clinical utility of measuring serum HO-1 over time for the purpose of the tracking disease activity in patients with AE, although we reported the AE of IPF case that the serial changes of serum HO-1 seemed to reflect disease activity of AE^33^. Fourth, as shown in the Supplement Fig. 1, although the number of cases was small, we demonstrated that serum HO-1 was significantly higher in AE than ARW due to infection, lung edema, and pneumothorax which was sometimes difficult to discriminate from AE. This result also needs further verification with more cases.

## Conclusions

In conclusion, serum HO-1 could be a clinically useful biomarker for the accurate diagnosis of patients with AEs.

## Supplementary Information

Supplementary Information 1.

Supplementary Information 2.

Supplementary Figure 1.

Supplementary Table 1.

## Data Availability

We have uploaded the raw data including IIPs and SIPs.
