# Supplementary material for: Assessment of diagnostic utility of serum hemeoxygenase-1 measurement for acute exacerbation of interstitial pneumonias
Source: Sci Rep. 2022 Jul 28;12:12935. doi: 10.1038/s41598-022-17290-0 (PMC9334264; doi:10.1038/s41598-022-17290-0)
Supplement: Supplementary file 1 — Supplementary Information 1. [file 41598_2022_17290_MOESM1_ESM.pdf]

Gender	Age	IIPs	AE or not
M	68	IIPS	AE
M	71	IIPS	AE
M	85	IIPS	AE
M	89	IIPS	AE
M	76	IIPS	AE
M	65	IPF	AE
M	78	IPF	AE
M	55	IPF	AE
F	73	IPF	AE
F	80	IPF	AE
M	79	IPF	AE
F	80	IPF	AE
M	79	IPF	AE
M	85	IPF	AE
M	76	IPF	AE
M	86	IPF	AE
M	80	IPF	AE
F	81	IPF	AE
M	85	IPF	AE
M	66	IPF	not AE
M	89	IIPS	not AE
M	81	IIPS	not AE
M	83	IIPS	not AE
F	83	IIPS	not AE
M	76	IIPS	not AE
M	71	IPF	not AE
M	75	IPF	not AE
M	63	IIPS	not AE
M	77	IIPS	not AE
M	83	IIPS	not AE
F	81	IIPS	not AE
F	73	IIPS	not AE
M	80	IIPS	not AE
M	69	IPF	not AE
M	57	IPF	not AE
M	76	IPF	not AE
M	63	IPF	not AE
M	72	IPF	not AE
F	80	IPF	not AE
M	77	IPF	not AE
M	84	IPF	not AE
M	86	IPF	not AE
M	71	IPF	not AE
M	83	IPF	not AE
M	76	IIPS	not AE

M	81	IIPS	not AE
M	82	IIPS	not AE
F	85	IIPS	not AE
F	79	IIPS	not AE
M	83	IPF	not AE
M	76	IPF	not AE
M	68	IPF	not AE
M	82	IPF	not AE
M	83	IIPS	AE
M	78	IPF	AE
M	84	IPF	not AE
F	71	IIPS	not AE
F	55	IIPS	not AE
M	78	IIPS	not AE
M	73	IPF	not AE
M	56	IPF	not AE
M	83	IPF	not AE
M	83	IPF	not AE
M	79	IIPS	not AE
M	73	IIPS	not AE
M	67	IIPS	not AE
F	66	IIPS	not AE
M	70	IPF	not AE
M	83	IPF	not AE
F	77	IIPS	AE
F	76	IIPS	AE
M	76	IIPS	AE
M	70	IPF	AE
M	77	IPF	AE
M	72	IPF	AE
M	68	IIPS	not AE
F	77	IPF	not AE
M	72	IPF	not AE
M	73	IIPS	not AE
M	55	IPF	not AE
M	68	IPF	not AE
M	71	IPF	not AE
M	64	IIPS	not AE
M	75	IIPS	not AE
M	71	IPF	not AE
M	68	IIPS	AE
M	75	IIPS	AE
M	74	IIPS	AE
M	53	IIPS	AE
M	52	IIPS	AE

AE or ARW or stable	CCI	GGO score	honeycomb score	HO-1 D1
AE	4	8	0	22.86
AE	2	14	0	33.34
AE	4	9	0	30.08
AE	2	11	0	17.84
AE	1	1	4	26.18
AE	2	16	7	129.6
AE	6	9	3	64.8
AE	3	14	10	104.2
AE	9	8	8	47.63
AE	1	8	5	156.2
AE	3	8	8	53.4
AE	3	11	2	53.53
AE	3	6	9	41.6
AE	3	11	8	51.8
AE	2	3	11	83.2
AE	5	4	8	25.46
AE	2	8	4	44.08
AE	2	22	3	26.88
AE	1	5	3	43.24
ARW	4	4	5	59.72
ARW	2	8	0	10.38
ARW	3	5	0	20.64
ARW	1	7	0	57.3
ARW	1	0	2	18.32
ARW	1	1	4	16.5
ARW	2	10	3	28.04
ARW	2	3	5	25.72
stable	0	3	0	26.82
stable	1	2	0	25.58
stable	1	2	0	28.76
stable	0	2	0	9.44
stable	0	5	0	8.64
stable	2	2	0	10.44
stable	2	3	5	18.78
stable	3	4	15	14.19
stable	3	3	13	19.83
stable	3	9	11	30
stable	3	2	5	30.74
stable	7	5	8	18.13
stable	1	3	5	10.84
stable	5	2	10	15.78
stable	1	6	4	9.66
stable	2	8	2	26.04
stable	3			15.24
stable	4	0	0	31.48

stable	3	2	0	13.6
stable	0	0	1	37.28
stable	2	3	3	0
stable	0	0	0	12.66
stable	4	2	5	16.32
stable	1	4	4	13.32
stable	2	2	7	18.68
ARW	3	1	3	9.54
AE	6	11	0	76.02
AE	9	12	4	28.24
ARW	4	18	9	33.74
stable	2	2	2	23.62
stable	4	4	1	19.64
stable	7			17.72
stable	4	2	0	7.22
ARW	1			21.84
stable	3	4	6	11.1
stable	3	0	5	15.12
stable	4	3	0	5.36
stable	2	1	0	12
stable	2	1	2	7.54
stable	1	1	0	9.9
stable	3	3	2	6.22
stable	4	1	9	10.24
AE	3	7	9	19.92
AE	3	12	0	24.54
AE	7	7	3	25.3
AE	3	7	2	17.48
AE	3	7	6	35.16
AE	4	7	9	17.74
ARW	3	2	7	14.36
ARW	2	5	0	13.02
ARW	4	5	9	16.94
stable	8	2	0	62.42
stable	2	8	5	22.1
stable	3	2	7	6.66
stable	3	2	9	8.34
stable	2	3	3	13.18
stable	2	2	0	8.72
stable	4	2	9	5.84
AE	6	10	0	33.06
AE	1	15	0	44.2
AE	3	10	1	51.4
AE	2	3	0	24.52
AE	6	6	0	32.18

LDH	SPD	KL-6
324	358	219
353	141	353
201	76.7	306
261	217	337
167	38.6	177
615	471	2603
273	122	796
444	347	860
445	271	1527
275	1280	1821
347	231	644
643	318	2306
316	105	616
336	105	1056
410	21.1	251
250	94.2	663
294	991	925
634	203	1055
184	73.6	265
254	155	1128
336	122	202
184	160	147
300	124	260
144	133	437
146		222
319		410
221	275	302
193	267	1005
183	272	777
191	110	503
186	116	434
222	54.3	838
186		415
208	109.6	1882
166	84.2	278
248	262	949
222	339	333
253	190	611
336	169	2152
212	266	898
157	92.6	626
201		677
247		
193		1179
197		

203	52	187
215	147	896
206	308	790
226		207
198	42.6	1069
266	700	321
232		2270
191	87.2	357
402	649	1383
233	291	392
296		554
206	360	2015
191		577
195		
144	99.5	221
164	98.9	377
176	306	771
228	50.2	777
178		471
212		
192	192	1419
205	58.8	214
153	86.3	305
232		623
183	44.3	133
324	288	2781
244	296	406
258	105	1487
273	171	1066
192	73.3	866
272		
607	88.3	722
243	43.5	682
267		
177		1895
194		482
192	154	818
196		376
195	107	818
198	152	778
210		418
308		754
277	366	1479
176	29.3	202
233	299	4274
