# Supplementary material for: Assessment of diagnostic utility of serum hemeoxygenase-1 measurement for acute exacerbation of interstitial pneumonias
Source: Sci Rep. 2022 Jul 28;12:12935. doi: 10.1038/s41598-022-17290-0 (PMC9334264; doi:10.1038/s41598-022-17290-0)
Supplement: Supplementary file 2 — Supplementary Information 2. [file 41598_2022_17290_MOESM2_ESM.pdf]

Gender	Age	SIP	AE or satable or inf	CCI
F	75	CTD	AE	2
M	62	CTD	AE	2
F	70	CTD	AE	2
M	78	CTD	AE	1
F	66	CTD	AE	1
F	72	CTD	AE	3
M	85	CTD	AE	2
F	72	CTD	AE	3
F	68	CTD	not AE	3
F	70	CTD	not AE	3
F	76	CTD	not AE	2
M	77	CTD	not AE	3
F	80	CTD	not AE	6
M	69	CTD	ARW	6
F	39	CTD	not AE	1
F	80	CTD	not AE	2
F	60	CTD	not AE	1
M	69	CTD	not AE	5
F	79	CTD	not AE	1
F	81	CTD	not AE	6
M	65	CTD	not AE	6
M	65	CTD	not AE	4
M	32	CTD	not AE	4
F	73	CTD	stable	1
F	77	CTD	stable	1
F	82	CTD	stable	3
M	66	CTD	stable	5
F	67	CTD	AE	2
F	62	O	not AE	0
F	28	O	not AE	1
F	62	O	not AE	0
F	30	O	stable	0

GGO score	honeycomb score	HO-1 D1	LDH	SPD	KL-6
2	3	32.02	519	221	2931
7	8	58	255	307	2624
18	1	48.76	393	144	920
2	6	18.5	275	137	1547
17	0	65.18	394	242	569
6	3	22.78	209	207	1558
17	0	46.52	265	404	951
16	1	20.96	175	181	315
5	0	43	338	197	1449
5	0	81.42	507	185	1254
10	0	10.6	145	139	362
1	1	8.36	148		388
6	0	7.7	276	176	2570
1	0	30.26	170		300
5	0	18.64	188	114	607
3	2	20.54	209		524
2	0	19.78	180	166	921
2	1	40.96	289		868
5	4	11.62	179	427	4217
13	4	9.92	399		5447
3	5	16.02	246		738
4	3	30.12	328	212	901
18	0	17.08	270	269	8508
5	3	22	294	149	544
4	6	16.42	218		909
2	2	12.14	197		
2	6	13.08	270		581
8	8	41.8	192		
1	0	19.22	276		
2	4	16.98	126	170	1852
3	0	15.72	269		
0	1	17.18	181		1990
