# Supplementary figures and images for: Assessment of diagnostic utility of serum hemeoxygenase-1 measurement for acute exacerbation of interstitial pneumonias
Source: Sci Rep. 2022 Jul 28;12:12935. doi: 10.1038/s41598-022-17290-0 (PMC9334264; doi:10.1038/s41598-022-17290-0)

## Slide 1
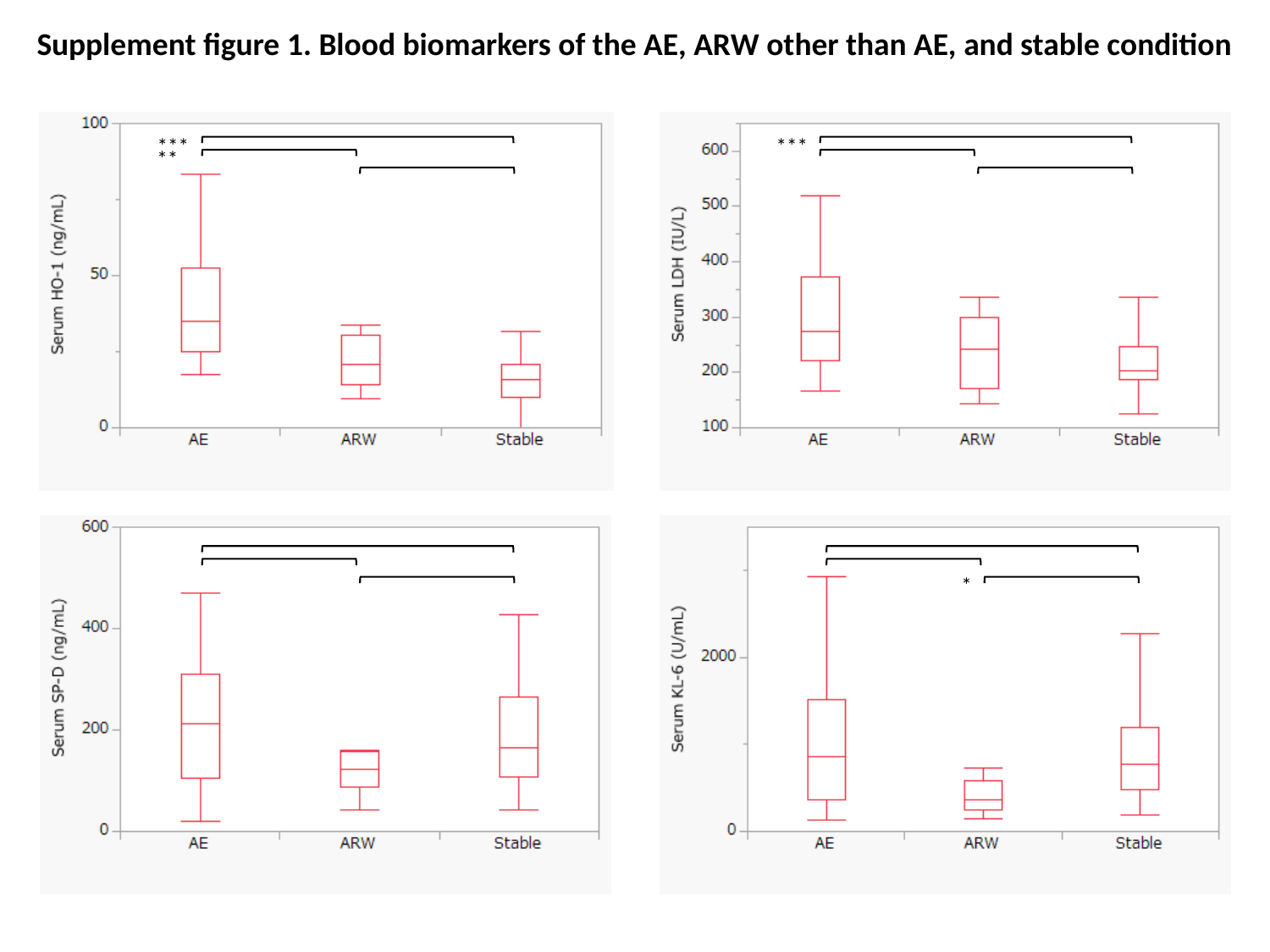

Supplement figure 1. Blood biomarkers of the AE, ARW other than AE, and stable condition
***
***
**
*

Supplement: Supplementary file 3 — Supplementary Figure 1. [file 41598_2022_17290_MOESM3_ESM.pptx]
