# Supplementary material for: Assessment of diagnostic utility of serum hemeoxygenase-1 measurement for acute exacerbation of interstitial pneumonias
Source: Sci Rep. 2022 Jul 28;12:12935. doi: 10.1038/s41598-022-17290-0 (PMC9334264; doi:10.1038/s41598-022-17290-0)
Supplement: Supplementary file 4 — Supplementary Table 1. [file 41598_2022_17290_MOESM4_ESM.pptx]

## Slide 1
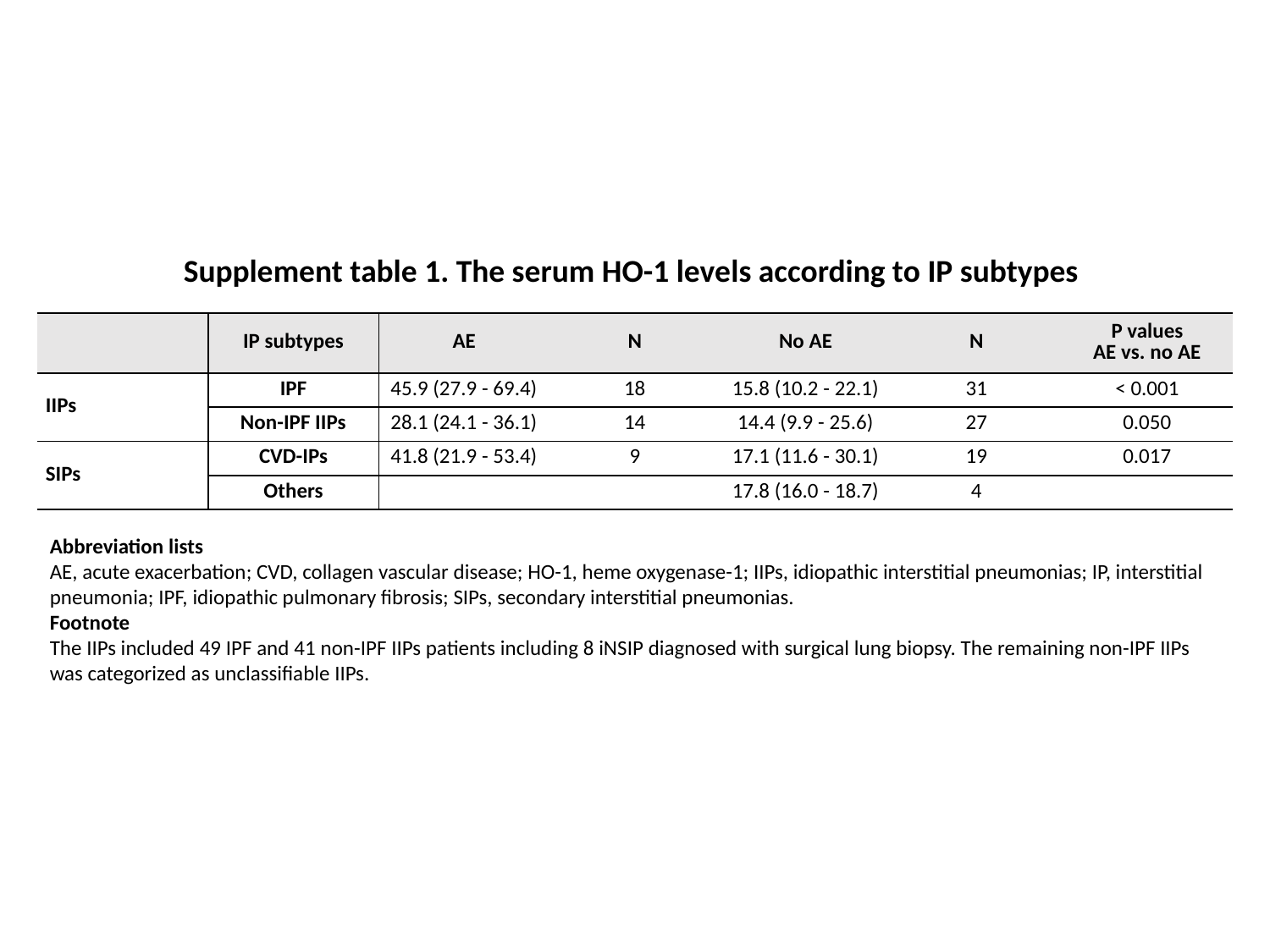

Supplement table 1. The serum HO-1 levels according to IP subtypes
| | IP subtypes | AE | N | No AE | N | P values AE vs. no AE |
| --- | --- | --- | --- | --- | --- | --- |
| IIPs | IPF | 45.9 (27.9 - 69.4) | 18 | 15.8 (10.2 - 22.1) | 31 | < 0.001 |
| | Non-IPF IIPs | 28.1 (24.1 - 36.1) | 14 | 14.4 (9.9 - 25.6) | 27 | 0.050 |
| SIPs | CVD-IPs | 41.8 (21.9 - 53.4) | 9 | 17.1 (11.6 - 30.1) | 19 | 0.017 |
| | Others | | | 17.8 (16.0 - 18.7) | 4 | |
Abbreviation lists
AE, acute exacerbation; CVD, collagen vascular disease; HO-1, heme oxygenase-1; IIPs, idiopathic interstitial pneumonias; IP, interstitial pneumonia; IPF, idiopathic pulmonary fibrosis; SIPs, secondary interstitial pneumonias.
Footnote
The IIPs included 49 IPF and 41 non-IPF IIPs patients including 8 iNSIP diagnosed with surgical lung biopsy. The remaining non-IPF IIPs was categorized as unclassifiable IIPs.
